# Real-world mastery of essential skills in a clinical research coordinator graduate program

**DOI:** 10.1017/cts.2025.10077

**Published:** 2025-07-07

**Authors:** Erika J. Stevens, Ginnette Watkins-Keller, Nancy Reilly, Reynold A. Panettieri, Barbara Tafuto

**Affiliations:** 1 MS CRM, Department of Health Informatics, School of Health Professions, Rutgers Health, The State University of New Jersey, Newark, NJ, USA; 2 Office of Human Research Services, Rutgers Cancer Institute of New Jersey, New Brunswick, NJ, USA; 3 Rutgers Biomedical Health Sciences Clinical Trials Office, New Brunswick, NJ, USA; 4 Rutgers Institute for Translational Medicine & Science, Child Health Institute of New Jersey, Rutgers Health, The State University of New Jersey, New Brunswick, NJ, USA; 5 New Jersey Alliance for Clinical Translation Science, Rutgers Health, The State University of New Jersey, New Brunswick, NJ, USA

**Keywords:** Experiential training, clinical research professional, academic clinical research, professional competencies, workforce development

## Abstract

Rutgers Health, Clinical Research Management (CRM) program with support from New Jersey Alliance for Clinical and Translational Science (NJ ACTS) provide scholarships to establish a “Clinical Research Experience” (CRE). The CRE focuses on building an entry-level Clinical Research Coordinator (CRC) workforce. The six-month precepted CRE is embedded in an accelerated master’s degree and demonstrates a skill-based approach to developing CRC resources. The CRE structure affiliated site collaboration; competency based curriculum objectives; standardized competency aligned on-boarding; and preceptor-evaluated performance. The experiential education is designed for academic medical centers (AMCs) to foster the development of qualified research coordinators. The CRE model supports “teach one, see one, do one” coupled with preceptor-evaluated feedback to cultivate clinical research competency.

## Introduction

The 2020 pandemic revealed a significant unmet need in training and recruitment of clinical research coordinators (CRC) and managers [1,2]. The level of required education and training for CRCs to reach competency is variable and challenges typical workforce development approaches [3]. New Jersey Alliance for Clinical and Translational Science (NJ ACTS), our CTSA Hub, identified a similar need for a skilled workforce due to institutional CRC vacancies across Rutgers Health. By developing and implementing a fast track, master’s level Academic Clinical Research Management (ACRM) degree, Rutgers Health addressed their unmet needs for expanding our workforce in research coordination. Our 16-month Master of Science program sits within the Clinical Research Management (CRM) program and includes an innovative approach to experiential education, aligning with the disparate needs of academic clinical research.

NJ ACTS, in collaboration with the Rutgers School of Health Professions (SHP), developed this clinical research experiential educational degree leveraging competencies within the Joint Task Force (JTF) for clinical trial competency, the clinical trial competency standard for clinical research professionals. This initiative aimed to develop CRCs through combined graduate-level education and hands-on experience in an expeditious manner.

## Background

While not a recognized occupation by the Bureau of Labor Statistics (BLS) [4], a BLS-issued special report focused described careers in biotechnology [5] and called attention to the role of the clinical research coordinator (CRC). The CRC role expanded exponentially and the continues to be essential for clinical research operations. As estimated, Career One Stop, sponsored by the U.S. Department of Labor, provides an occupation profile for the Clinical Research Coordinator and projects an 8% annual growth in job openings. Considered a “bright outlook” with a score of 100, clinical research coordinators are expected to grow beyond the average career growth [6]. This forecast suggests a need to increase the number of qualified clinical research coordinators.

Clinical research coordinators are critical to the conduct of clinical research activity at Academic Medical Centers (AMCs) [7]. The CRC serves as the liaison for the facilitation of clinical research activity [8]. The value of the CRC is evidenced in increase of participant enrollment and risk mitigation [9]. The tasks performed by CRCs are substantial, requiring training and acquired skills [10]. Inconsistencies across clinical research coordinators in academia created a lack of clarity on the required skills for job performance. This inconsistency in assigned tasks called for the standardization of skills.

The JTF established the framework and level set the criteria for clinical research competencies [11]. The eight domains within this framework synthesized the core facets of clinical research operational competencies for the field and established competency levels (basic, skilled, and advanced).

While the JTF-established competencies for clinical research are well recognized [12], uncertainty remains around the required level of education and experiential training needed to perform those tasks. A multitude of pathways exist for obtaining clinical research competencies. The industry standard for clinical research training relies on member-accredited certifying organizations (Association of Clinical Research Professionals (ACRP) [13] and Society of Clinical Research Associates (SoCRA) [14]. ACRP and SoCRA training opportunities provide clarity on responsibilities and supports employee retention; however, non-experiential training can fail to translate to effective workforce practices. ACRP and SoCRA are not designed to provide applied experiential training.

Micro-credentialing programs, such as badging and certifications is an alternative approach to developing competencies [15–17]. The value of micro-credentialing remains unknown given its nascent existence and assessment of clinical research skills in badging programs is challenging. The ACRP and SoCRA require a two year minimum industry experience prior to certification. This presents a major hurdle for those entering the profession [18]. NJ ACTS CRC offers a badge is designed to build a foundation for an understanding of CRC roles but cannot offer experiential training. Accordingly, the approach is designed for entry-level positions [19].

Conventional CRC training embraces traditional academic programs. As the clinical research industry expanded, so did the need for academic programs in clinical research. Nonetheless, education does not equate to experience and may not translate into clinical research operations on the job. The delicate balance of knowledge, experience and competency development within clinical research coordination remains unmet. Experiential education may be the common missing link to support this challenge.

SHP improved the balance of knowledge, experience and competency through the implementation of experiential education. Rutgers Health, NJ ACTS and the SHP MS CRM program developed and implemented an innovative approach to address this unmet need. Goals included build a fast track to fill gaps within the clinical research units, develop a comprehensive approach to academic clinical research, and align the competencies to the skilled and advanced experiential levels as outlined by JTF.

## Materials and methods

The Clinical Research Experience (CRE) workforce development curriculum is intentionally designed to prepare clinical research workforce for career entry into AMCs. The Rutgers MS CRM in collaboration with the Rutgers Cancer Institute [20] and support from RBHS Clinical Trials Office (CTO) [21], operationalized clinical research mentored rotations, developed JTF competency aligned educational objectives and implemented JTF experiential activities.

Leveraging existing MS CRM core curriculum and newly developed academic research specific courses, students participate in a 16-month full-time hybrid master program through the ACRM Track. This track covers the theory and application of research methods and statistics, regulatory and legal governances over clinical trials performed at AMCs, diseases and pharmacological treatment, clinical research operations, and project management. As part of this hybrid, full-time experiential track, the hands-on didactic portion is offered online and in-person. The CREs are threaded throughout the program. For example, in “Clinical Research Operations from an Academic Perspective” course, students observe real-time, study intake, scientific review, human research oversight, recruitment facilitation and study implementation activities. *See Table 1: MS ACRM RG*

**Table 1.** Masters of science in clinical research management academic clinical research management requirements for graduation

Rutgers School of Health Professions Requirements for Graduation for the Program of MASTERS OF SCIENCE IN CLINICAL RESEACRH MANAGEMENT ACADEMIC CLINICAL RESEARCH MANAGEMENT		
**COURSE CODE**	**TITLE**	**CREDIT**
	**FALL-1**	
BPHE 5200	Medicines Development	3
BPHE 5310	Scientific Concepts and Research Design	3
BPHE 5510	Overview of Disease Process & Treatment	3
BPHE 5521	Regulatory and Ethical Requirements in Clinical Investigations	3
	**SPRING-1**	
BINF 5075	Clinical Trial Data Management	3
BPHE 6459	Clinical Research Operations from an Academic Perspective	3
BPHE 6830	Project Management and Leadership	3
	**SUMMER-1**	
BPHE 6200	Concepts of GxPs and Quality Assurance	3
BPHE 6929	Clinical Research Experience I	3
	**FALL-2**	
BPHE 6939	Clinical Research Experience II	3
BPHE 6800	Regulatory Writing for Submissions and Publications	3
BPHE 6985	Patient Engagement	3
TOTAL		36^*^

* Student must fulfill 36 credit requirements in order to graduate.

In addition to course work, students complete over 500 hours of on-site clinical research educational experiences that provides them with hands-on training in the conduct of clinical trials with supervision and feedback. Students are placed in CREs based on their background, aligned to preference and are offered experiences in one of the operational pillars of Clinical, Administrative, and Regulatory/Compliance. In each of the areas, the students are provided opportunities to develop competencies and obtain skills that will help them in future employment opportunities.

The CRE structure includes the following facets:

1. Affiliated site collaboration;
2. Competency-based curriculum objectives;
3. Standardized competency aligned on-boarding; and
4. Preceptor evaluated performance.

### Affiliated site collaboration

Through system affiliated connections, the MS CRM Director, CRE, collaborated with leaders of two key clinical research support service providers; the Office of Human Research Services (OHRS) and the CTO. Site leaders collaborate to identify preceptors and to align operational objectives for the clinical research experiential placements (CRE I/II, as identified in Table 1).

As part of Rutgers Health, Rutgers Cancer Institute and Robert Wood Johnson (RWJ) Barnabas Health, the OHRS provides support including, but not limited to: research protocol and consent submission, pre-study activities, activation/ startup, screening, consent and eligibility verification, enrollment support, quality assurance, regulatory and data management. Also, OHRS administratively oversees the Rutgers Cancer Institute Human Research Oversight Committee (HROC) and the Scientific Review Board (SRB). This collaboration provides learners with virtual invitations to observe HROC and SRB meetings. For this reason, Rutgers Cancer Institute (National Cancer Institute (NCI) designee) affiliated site collaboration is able to provide students with CRE precepted rotations across three locations in New Jersey (New Brunswick, Newark and Hamilton).

Developed under the Rutgers Institute for Translational Medicine and Science (RITMS), with support from NJ ACTS and Rutgers Health, the Rutgers Clinical Trial Office provides centralized administrative functions to support clinical research. Core services of the Rutgers CTO include but are not limited to: feasibility assessment, contract and budget negotiation, research business system solutions, regulatory technical assistance, and recruitment support. The CTO supports non-oncology clinical research, facilitating study intake review and clinical research start-up activities. The CTO provides students with virtual invitations to observe new study and start-up meetings. The CTO affiliated site collaboration provides a select number of students with research technology focused CRE precepted rotations.

### Competency-based curriculum objectives

The CRE spans two semesters and offers learner distinct exposure to apply CRC skills. Learners participate in managing a clinical trial, such as protocol activation, eligibility screening, quality assurance or data management. *See Appendix 1: CRE I/II Course Objectives*

As mapped to the JTF, clinical research professional activities span eight domains, capturing the skillset required for competency. For example, students may review original source documentation to confirm eligibility or assist preceptors in obtaining eligibility requirements for potential patients who may enroll to trials. In pulling source documentation, ACRM students assist in answering eligibility questions identified on the eligibility checklist. Leveraging the skills gained in the core curriculum, such as analyses of protocol requirements, students align the criteria to an eligibility checklist reviewed by their preceptor. These interactions provide the students with collaborative interfacing with clinical research professionals actively working in clinical research.

**Figure 1.**
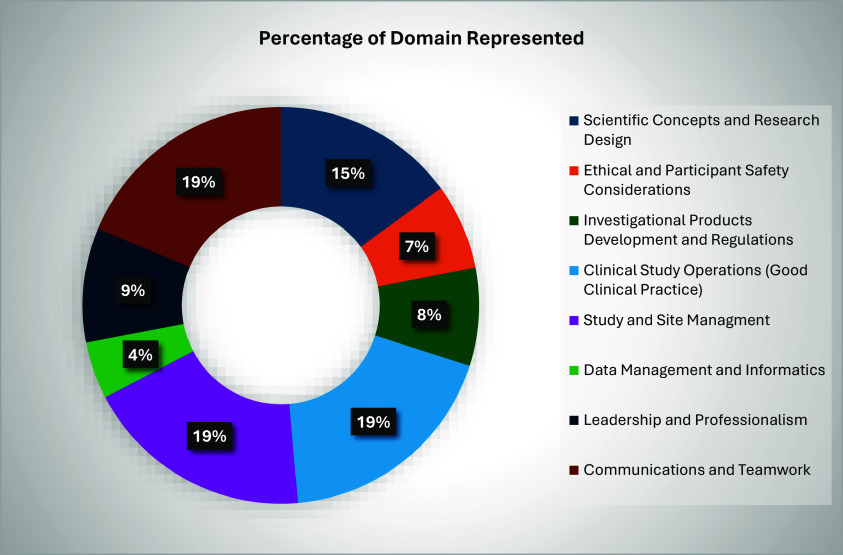
Clinical research experience (cre) skilled application to JTF.

The objectives of the CRE I/II align to the JTF for Clinical Trial Competency. *See Figure 1: Clinical Research Experience (CRE) Skilled Application to Joint Task Force (JTF) for Clinical Trial Competency (JTF)*

The CRE I/II rotations include skilled-level application across all eight domains. The CREs heavily emphasize domain four, *Clinical Study Operations (GCP)*, including study management tasks across all ten leveled core competency skills (4.1–4.10), domain five, *Study and Site Management* operations across all seven core competencies (5.1–5.7) and domain eight, *Communications and Teamwork* across all four of the leveled competencies at the site level. See examples from each domain:

Domain 1: *Scientific Concepts and Research Design –* During review of protocols at site placements, students assess and align primary and secondary endpoints for measuring study outcomes.

Domain 2: *Ethical and Participant Safety Concerns –* As part of the pre-screening processes, students review eligibility checklists aligned to protocol objectives and confirm appropriate application. This study related exercise determines potential eligibility of study participants. Application of clinical trial activities include lab processing, serving as witnesses during the informed consent (IC) processes to demonstrate safeguards and supporting data quality following audits with identified eligibility deviations requiring next steps.

Domain 3: *Investigational Products Development and Regulation –* Learners assist investigators with site files for regulatory review, support preceptors in assigning adverse event grading and review charts leveraging the investigational brochure (IB) for risks associated with the study trial.

Domain 4: *Clinical Study Operations –* Students describe the role of the IRB as per regulations and apply appropriate privacy safeguards related to the conduct of assigned study activity. Additional activities include assistance with audits for GCP compliance, support of serious adverse event (SAE) reporting and drafting corrective and preventative actions (CAPA).

Domain 5: *Study and Site Management* – To demonstrate strategies for managing participant recruitment, students track study related recruitment trends and identify opportunities to leverage technology solutions to bolster recruitment. Learners evaluate feasibility of potential studies and create recruitment plans leveraging artificial intelligence (AI) solutions.

Domain 6: *Data Management and Informatics –* Students review source documents for quality review and reconciliation of data entry errors.

Domain 7: Leadership and Professionalism – Throughout the experiential rotations students maintain professionalism with preceptor interface and comply with site processes, procedures and policies.

Domain 8: *Communications and Teamwork* – Progress updates on assigned clinical research activities are presented by learners during weekly department and/or specific disease group meetings Leveraging evidence based literature analyses and synthesis of experiential placement activities, students present results in live discussions.

Applying the knowledge learned through JTF aligned CRM graduate course curriculum, ACRM students participate in a variety of operational activities. Direct involvement and implementation of theoretical knowledge provides students with skilled-level competency. Leveraging a structured approach for student on-boarding provides consistency to support placement success.

### Standardized competency aligned on-boarding

Coupled with CRE course requirements, standardized competency aligned on-boarding is required during the experiential placements. Students complete the NJ ACTS CRC Level 1 badge, CITI e-learning [22] encompassing 15 topic areas and CRE standardized on-boarding criteria. *See Appendix II: CRE Onboarding Checklist*

The CRE clinical operations orientation provides students with consistent operational management insight into systems, processes and administrative functions. This orientation offers students a concrete and measurable criteria for comprehensive support. Throughout the CRE I/II rotations, students receive real-time preceptor feedback.

### Preceptor evaluated performance

In similar health sciences related professions, such as occupational therapy, clinical rotations, including “see one, do one, teach one,” [23,24] require a component for competency and mastery success. The CRE approach by the MS CRM follows this model. Students are assigned a primary site preceptor as a mentor and evaluator of student performance. Site preceptors provide students with feedback throughout the CRE rotations. Students confirm within the learning management system (LMS) to establish weekly preceptor meetings and receive documented evaluations at three intervals per semester. The first evaluated feedback provides the initial alignment of student activities to placement objectives detailed within the week-#2 progress report. The second 360 feedback is student summarized, which leverages preceptor feedback and self-assessment. The final performance evaluation utilizes a ranked competency scale assessing student performance at the conclusion of each CRE semester.

### Next steps

Combining graduate-level education with precepted experiential learning facilitates skills development and suggests improved competency for entry-level clinical research coordinators. This applied educational model moves beyond the JTF fundamental level and provides the learner with direct application of skills. Rather than simple simulation, the CRE applies theoretical knowledge at the skilled level. While traditional approaches to gaining JTF Competencies through coursework and simulations have proven to be successful overtime over time, the competences fall short at the fundamental level. As described, the comprehensive experiential program outlined offers a robust, potentially scalable and expeditious opportunity for graduate students to gain real-world mastery of essential skills for a future career in clinical research.
